# Application of Ionic Liquids in the Microwave-Assisted Extraction of Pectin from Lemon Peels

**DOI:** 10.1155/2012/302059

**Published:** 2012-03-05

**Authors:** Huang Guolin, Shi Jeffrey, Zhang Kai, Huang Xiaolan

**Affiliations:** ^1^Key Laboratory of Radioactive Geology and Exploration Technology Fundamental Science for National Defense, East China Institute of Technology, Fuzhou City, Jiangxi 344000, China; ^2^School of Chemical and Biomolecular Engineering, The University of Sydney, Sydney, NSW 2006, Australia

## Abstract

Microwave-assisted extraction of pectin from lemon peels by using ionic liquid as alternative solvent was investigated. The extracted pectin was detected by Fourier transform infrared spectra. The extraction conditions were optimized through the different experiments in conjunction with the response surface methodology. A pectin yield of 24.68 % was obtained under the optimal parameters: the extraction temperature of 88°C, the extraction time of 9.6 min, and a liquid-solid ratio of 22.7 ml · g^−1^. The structure of the pretreated lemon peel samples and the samples after microwave-assisted extraction were characterized by a field emission scanning electron microscope.

## 1. Introduction

A significant large amount of lemon peels have been disposed as an industrial waste each year. It is not only a substantial burden to the environmental protection, but also a tremendous waste since lemon peels are composed of many valuable ingredients. Pectin, one of the ingredients, exhibits great biological and pharmacological potentials including antibacterial, hemostasis, detumescence, detoxification, antiobesity, and so on [1–3]. It has been estimated that about 5000 tons of pectin could be produced each year if there is a feasible extraction technology. In recent years, pectin has been widely used in the food, petroleum, chemical, electronics, and pharmaceutical industries [4, 5]. It is, therefore, of great interest to develop a new technology not only to ease the environmental burden but also to turn waste into useful products [6].

 There were several conventional pectin extraction methods, such as boiling water extraction, acid extraction, ion exchange resin extraction, microbiological assay, and ammonium oxalate extraction [7–12]. These techniques  are  general tedious,  time-consuming,  labor-intensive, and/or involve a bulk amount of volatile/hazardous organic solvents or strong acid-base. Recently, microwave-assisted extraction (MAE) has been developed, which can directly extract bioactive compounds from different solid matrixes [13, 14]. Although this MAE approach is rapid and effective, the organic solvents and strong acid or base used in the extraction are toxic, volatile, flammable, or corrosive. Such process is neither sustainable nor environmental friendly.

 Ionic liquids, neoteric solvents composed of organic cations and inorganic or organic anions, have unique excellent characteristics in extracting and microwave-absorbing ability [15]. Being efficient and environmental friendly extraction solvents, they have potential applications in many fields of the chemistry industry, especially in material separation. In fact, ionic liquids have been successfully applied as alternative solvents in the microwave-assisted extraction of bioactive substances for several years [16–18].

In our previous study, effects of the extraction parameters such as extraction temperature, extraction time, and extraction solvent volume were investigated [19]. Hence, a further attempt of determination of sample microstructures and extraction mechanism was approached. The subsequent work studies the potential applications of ionic liquids as alternative solvents to extract pectin from lemon peels assisted by microwave. The response surface methodology (RSM) is used to analyze the impact of the processing parameters and to optimize these parameters systematically afterwards. The ionic liquids-based microwave-assisted extraction approach proposed here is then compared with conventional extraction methods. Fourier transform infrared (FTIR) and scanning electron microscopy (SEM) technologies are used to study the sample microstructures before and after extraction.

## 2. Experimental

### 2.1. Chemical Composition of Lemon Peels

The lemons were purchased from a local supermarket, Fuzhou, China. The minced lemon peels were treated by adding 3.5 times the volume of water and then placed in a microwave radiation for 15 min to remove soluble sugar and pigment. The treated lemon peels were then dried, milled, sieved (80-mesh stainless steel screen), and stored in closed desiccators at 4°C before use.

Typical chemical compositions of the treated lemon peels were analyzed by China National Standards (GB), and the results are as follows: a moisture content of 11.15 ± 0.37% (w/w) (GB/T 5009.3-2003), an ash content of 3.66 ± 0.04% (w/w) (GB/T 5009.4-2003), a protein content of 3.16 ± 0.25% (w/w) (GB/T 5009.5-2003), a fat content of 1.52 ± 0.22% (w/w) (GB/T 5009.6-2003), a total carbohydrate content of 14.03 ± 0.79% (w/w) (GB/T 15672-1995), a crude fiber content of 9.50 ± 2.09% (w/w) (GB/T 5515-1985), a pectin content of 27.02 ± 0.5% (w/w) (NY 82.11-1988), and a caloric value (C.V) of 78.85 cal which was calculated by the following expression (1):

(1) C.V·(calories/100 g)=P×4.0+F×9.0+C×3.75,

where *P*, *F*, and *C* are the contents in g per 100 grams of protein, fat, and carbohydrates, respectively.

### 2.2. Preparation of Ionic Liquids

The ionic liquids in this study were 1-butyl-3-methylimidazole chloride ([Bmim]Cl), 1-butyl-3-methylimidazolium bromide ([Bmim]Br), 1-butyl-3-methylimidazolium tetrafluoroborate ([Bmim][BF_4_]), 1-ethyl-3-methylimidazolium bromide ([Emim]Br), 1-ethyl-3-methylimidazolium tetrafluoroborate ([Emim][BF_4_]), and 1-allyl-3-methylimidazolium chloride ([Amim]Cl). They were synthesized based on references [20, 21] and fully characterized by FT-IR and ^1^H-NMR. The spectra of ionic liquids are basically in consistence with those in the references. Before use, 1-methylimidazole, 1-chlorobutane, 1-bromobutane, bromoethane, and allyl chloride were purified by vacuum distillation and carbazole was purified by sublimation. All reagents were of analytical grade. The solutions were prepared with the corresponding ionic liquids in distilled water.

### 2.3. Microwave-Assisted Extraction Procedure

**Figure 1:**
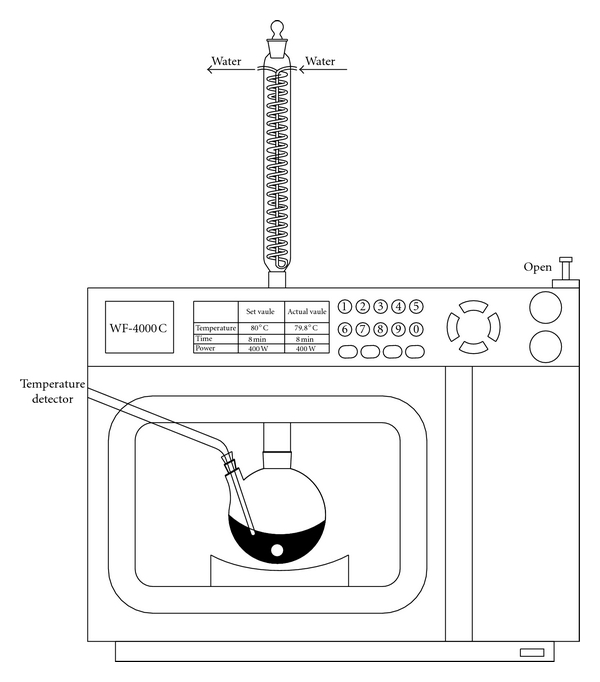
The apparatus of microwave reaction system.

The extraction process was carried out in a WF-4000C microwave reaction system (Shanghai EU Microwave Chemistry Technology, China) equipped with a temperature control system, a magnetic stirrer and a water condenser. The schematic diagram of the pectin extraction is shown in Figure 1. One gram of accurately weighed pretreated peel sample was mixed with 15 mL of various ionic liquids solutions, and then fed into the extraction apparatus. Extraction process took place at the temperature of 80°C for 5 min. Following this extraction process, the extracts obtained were filtrated and then diluted to fixed volume with deionizer water for subsequent spectra analysis. The extraction yield of pectin was defined as follows:

(2) $$\begin{array}{cc} & \text{yield}\;\text{of}\;\text{pectin}\;\left(\%\right)\\ & \;\;\;\;\;\;=\frac{\text{the}\;\text{mass}\;\text{of}\;\text{pectin}\;\text{in}\;\text{extraction}}{\text{the}\;\text{mass}\;\text{of}\;\text{pectin}\;\text{in}\;\text{sample}}\times 100. \end{array}$$

### 2.4. Separation and Purification of Pectin

Each extracted product from the experiments was filtered to remove peel pulp and then was concentrated by evaporation to one fifth of the initial volume with a rotary evaporator (RE 201D, Shanghai, China). Pectin was precipitated from the extraction solution by the addition of the equal reduced volume of 96% (w/w) ethanol. The mixture of solvent and precipitate was stirred for 10 min and then kept stationary for 2 h. After stripping of the ethanol, the ionic liquids can be recycled. The coagulated pectin was washed with 70% (w/w) ethanol for three times. The product of pectin was dried at 40°C. The yield of pectin was calculated on the basis of the dry weight and expressed as a percentage.

### 2.5. Analysis of Pectin

The Fourier transform infrared spectra was recorded for the pectin extracted in the region of 400–4000 cm^−1^ on an FT-IR spectrometer (Nicolet 380 model, Thermo Fisher Scientific, USA) by using pressed KBr pellets. The microstructures of the lemon peels before and after extraction were studied by the field emission scanning electron microscope (SEM) (JSM-6700F, JEOL, Japan), which was operated at 10 keV and at accelerating voltage of 5 kV. The pieces of dried samples were glued to a copper holder using platinum conducting paint.

### 2.6. Central Composite Design

Optimizing a process with many parameters and these parameters having interaction are a trivial task. In chemical or engineering processes, industrial research and biological investigations, the response surface methodology (RSM) is a popular and effective optimization tool [22–24]. The RSM has a main advantage of that it can reduce the number of experimental trials needed to evaluate multiple parameters and their interactions. The present study employs the RSM along with a central composite design for optimization. According to Zhang [25], the total number of experimental combination is 2^*k*^ + 2*k* + *n*_0_, where *k* is the number of independent variables and *n*_0_ is the number of repetitions of the experiments at the centre point. For statistical calculation, the experimental variables *X*_*i*_ have been coded as *x*_*i*_ according to the following transformation equation (3):

(3) $$x_{i}=\frac{X_{i}-X_{0}}{\delta X},$$

where *x*_*i*_ is the dimensionless coded value of the variable *X*_*i*_, *X*_0_ is the value of *X*_*i*_ at the center point, and *δX* is the step change.

A preliminary study on the single factors showed that the extraction temperature, the extraction time, and the liquid-solid ratio were the main parameters affecting the yield of pectin extraction. Based on the study, a three-variable, five-level Box-Behnken design was applied to optimize the extraction condition for the pectin yield from lemon peels. Three independent variables were extraction temperature (*X*_1_), extraction time (*X*_2_), and liquid-solid ratio (*X*_3_). The factors in this study are listed in Table 1. The yield of pectin was analyzed by multiple regression through the least squares method to the quadratic response surface model as follows:

(4) $$Y=\beta_{0}+\overset{3}{\underset{i=1}{\sum }}\beta_{i}x_{i}^{2}+\overset{3}{\underset{i=1}{\sum }}\beta_{ii}x_{i}^{2}+\overset{5}{\underset{i=1}{\sum }}\overset{3}{\underset{j=i+1}{\sum }}\beta_{ij}x_{i}x_{j},$$

where *Y* is the predicted response variable; *β*_0_, *β*_*i*_, *β*_*ii*_, and *β*_*ij*_ are constant regression coefficients for intercept, linear, quadratic, and interaction terms, respectively; *x*_*i*_ and *x*_*j*_ are the independent variables.

### 2.7. Statistical Analysis

To obtain the coefficients of the quadratic polynomial model, the responses obtained from the experimental design set (Table 2) were subjected to multiple nonlinear regression using the software Design-Expert 7.1 Trial (State-Ease, Inc., USA). The quality of the fitted model was expressed by the coefficients of determination *R* ^2^, and its statistical significance was checked by an *F* test.

## 3. Results and Discussion

### 3.1. Determination of Ionic Liquids

Various ionic liquids ([Bmim] Br, [Bmim] Cl, [Emim] Br, [Bmim][BF_4_], [Emim][BF_4_], and [Amim] Cl solutions) as alternative solvents under the same microwave-assisted extraction conditions have been evaluated. A pectin yield of 15.7%, 19.91%, 10.8%, 11.2%, 7.8%, and 12.1%, respectively, has been achieved. The results show that the highest yield of pectin extraction can be obtained when [Bmim] Cl has been used. The probable reasons were that [Bmim] Cl increased the solubility of pectin in solutions due to its multi-interactions between pectin molecular and the ionic liquid, especially hydrogen bond. Moreover, ionic liquids can effectively increase dissipation factor of solution and the transfer rate of microwave energy to samples which can affect extraction efficiency. The highest extraction efficiency of [Bmim] Cl can be explained by the fact that [Bmim] Cl contains imidazolium cation and chlorine anion. The intermolecular hydrogen bonds between chlorine anion and hydroxyl of pectin can be easily formed, thereby increasing extraction yield. 

**Figure 2:**
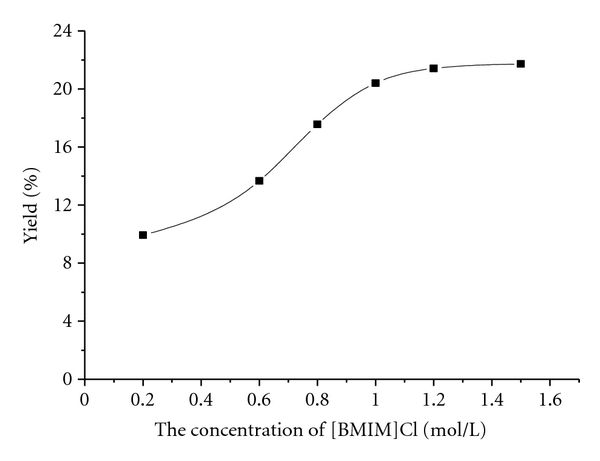
Effects of [Bmim] Cl concentration on the yield of pectin.

The effect of the ionic liquids concentration on the yield of pectin was investigated in the range of 0.2 to 1.5 mol·L^−1^, while the extraction time was fixed at 5 min, the extraction temperature was 80°C and the liquid-soild ratio remained at 15 mL·g^−1^. The result was plotted in Figure 2. 

As shown in Figure 2, the yield of pectin increased gradually as the increase of the [Bmim] Cl concentration over the range of 0.2 to 1.0 mol·L^−1^. It reached the maximum of 21.6% at the [Bmim] Cl solution concentration of 1.0 mol·L^−1^. As [Bmim] Cl solution concentration increased further, the yield of pectin remained constant, indicating the [Bmim] Cl solution concentration higher than 1.0 mol·L^−1^ gives no additional benefit. Hence, the most suitable [Bmim] Cl concentration for the pectin extraction is 1.0 mol·L^−1^. 

### 3.2. Optimization of MAE Conditions Using RSM

#### 3.2.1. Regression Models of Response 

By applying multiple regression analysis on the experimental data, the following second-order polynomial equation was found to be able to interpolate the pectin production regardless of the significance of coefficients:

(5) $$\begin{array}{cc} Y\; & =21.85594+2.87416\;x_{1}+0.88400\;x_{2}+0.93762\;x_{3}\\ & \;-0.12875\;x_{1}x_{2}-0.096250\;x_{1}x_{3}+0.38625\;x_{2}x_{3}\\ & \;-0.59548\;x_{1}^{2}\;-0.68564\;x_{2}^{2}\;-0.92428x_{3\;}^{2}, \end{array}$$

where *Y* is the predicted response (yield of pectin), and *x*_1_, *x*_2_, *x*_3_ are coded values of extraction temperature, extraction time, and liquid-solid ratio, respectively. 

The statistical significance of (5) was checked by *F* test, and the analysis of variance (ANOVA) for the response surface quadratic model is showed in Table 3. 

The analysis of variance (*F* test) shows that the second-order model matches well with the experimental data. The coefficient of variation (CV) indicates the degree of the precision to which the treatments are compared. Here, a lower value of CV (3.12) indicates the experiments are more precise and reliable. The precision of a model can be represented by the determination coefficient (*R* ^2^). The determination coefficient (*R* ^2^) implies that the sample variation of 98.20% for the yield of pectin is attributed to the independent variables, and only about 1.80% of the total variation cannot be explained by the model. 

The *P*-values represent the significance of the corresponding coefficients in terms of the pectin yield, with a smaller *P*-values indicating more significant impact of the corresponding coefficient. The *P*-values less than 0.05 indicate the model terms are significant, whereas the *P*-values greater than 0.1 demonstrate that the model terms are insignificant. As can be seen from the Table 3, among the independent variables, *X*_1_ (extraction temperature), *X*_2_ (extraction time), and *X*_3_ (liquid-solid ratio) have a significant effect on the yield of pectin. Thus, the response is sufficiently explained by the model. 

#### 3.2.2. Analysis of the Response Surface

**Figure 3:**
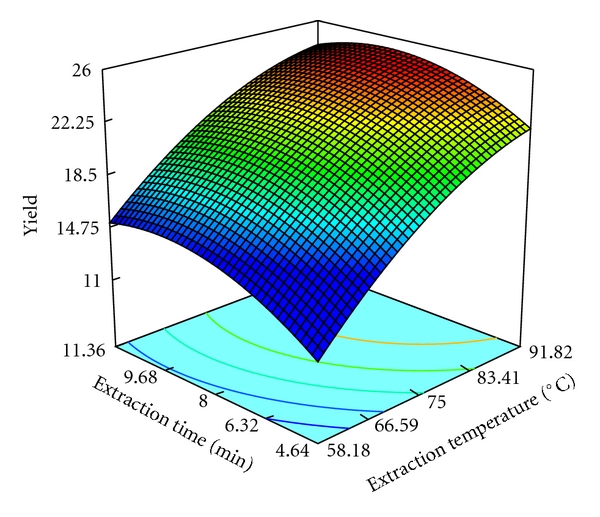
Response surface and contours of the effect of extraction time and temperature on the yield of pectin.

**Figure 4:**
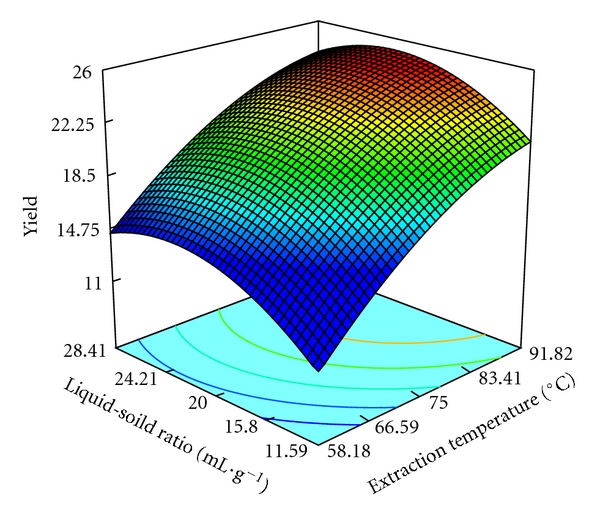
Response surface and contours of the effect of liquid-solid ratio and extraction temperature on the yield of pectin.

**Figure 5:**
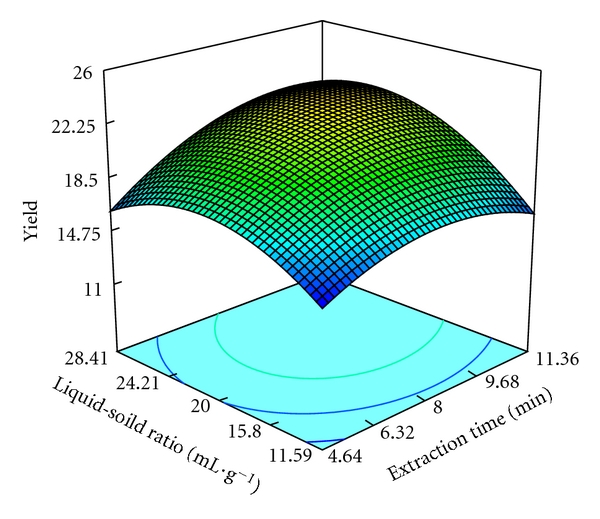
Response surface and contours of the effect of liquid-solid ratio and extraction time on the yield of pectin.

The three-dimensional response surface are plotted and illustrated in Figures 3–5. The vertical axe represents the extraction yield of pectin (%), and the two horizontal axes represent any two of the three independent variables. The topography of these response surfaces is also illustrated by the response contour lines in two variable planes. 

As can be seen from these response surfaces, the extraction temperature, the extraction time, and the liquid-solid ratio affect the pectin yield significantly. The response contour lines at bottom of the three-dimensional plot are more circular, and the interaction between two factors is not remarkable. From these figures, only the interaction between liquid-solid ratio and extraction time is significant. The three-dimensional plot of interaction between extraction time and temperature indicates the maximum extraction yield of pectin is achieved at the extraction temperature between 83.14°C and 91.82°C, and the yield is increased with the increase of the extraction time from 4.64 min to 9.68 min (Figure 3). Liquid-solid ratio affected the extraction yield of pectin more significantly than extraction time (Figure 4). It shows the increase of the extraction yield as the liquid-solid ratio is increased from 11.59 to 24.21 mL·g^−1^. When comparing Figure 3 with Figure 4, they are very similar. As can be seen from Figure 5, it indicates extraction time and liquid-solid ratio affected the extraction yield of pectin more significantly. The optimum liquid-solid ratio and extraction time are located between 15.90–24.21 mL·g^−1^ and 8–10 min, respectively. 

#### 3.2.3. Determination of Optimal Conditions

Based on the fitting model by the design expert 7.0 software, the optimal conditions are predicted to be the temperature of 88.36°C, the extraction time of 9.63 min, and the liquid-solid ratio of 22.71 mL·g^−1^. Under these conditions, the expected value of the pectin yield is 25.10%. The predicted optimum conditions were verified by 3 parallel experiments. Considering the actual experimental settings, the experiments were carried out at the following conditions: the extraction temperature of 88°C, the extraction time of 9.6 min, and the liquid-solid ratio of 22.7 mL·g^−1^. The resulted actual pectin yield was 24.68%, which agrees well with the expected value of 25.10%. 

### 3.3. Comparison of Microwave-Assisted Extraction with Conventional Extraction

For comparison, heat reflux extraction (HRE) method was also selected as the reference method for the extraction of the pectin. A water-bath was performed with a 1.0 g sample and 22.7 mL 1 mol·L^−1^ [Bmim] Cl in a flask and the suspensions were boiled for 2 h. Compared with HRE method (88°C, 2 h, pectin yield of 12.73%), the MAE method had higher extraction efficiency (88°C, 9.6 min, pectin yield of 24.68%). In MAE method, microwaves directly heat solvents and the sample, while in HRE method a finite period of time is needed to heat the vessel before the heat is transferred to solvents and the sample. The direct interaction of microwaves with the ionic liquids solutions and free water molecules present in the cells results in the subsequent rupture of the cells and release of intracellular products into the solvent. So the microwave assisted extraction can achieve higher extraction efficiency by using less solvent at shorter extraction time. 

### 3.4. SEM Scan Analysis of Extracts

**Figure 6:**
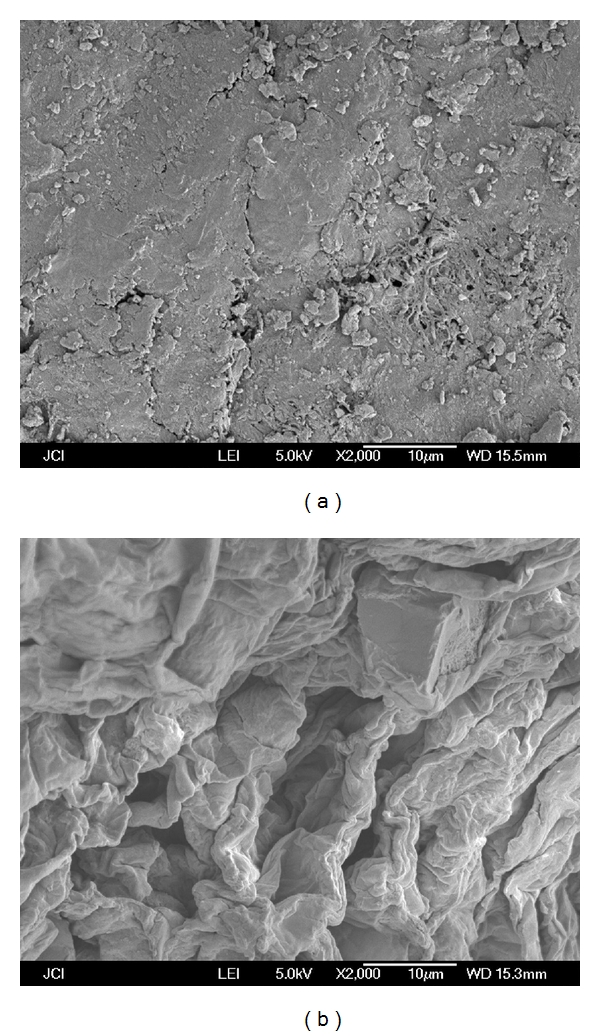
Scanning electron micrographs of lemon peels: pretreated lemon peels (a); after MAE for 10 min (b).

In order to understand the extraction mechanism, the structure of the pretreated peel sample and the peel sample after the MAE were studied by SEM. The results are depicted in Figure 6. It is clear that the structure of the pretreated peel sample before MAE remains intact (Figure 6(a)) whereas the structure of the samples after MAE is loosen (Figure 6(b)), indicating the microstructures are destroyed by MAE significantly. It confirms that the pectin released from lemon peels. The mechanism can be explained as follows: With the absorption of the microwave, the cell's temperature of the lemon peels increases sharply, and the pressure in the cells exceeds the limit. As a result, the cells split rapidly and the peel tissues open up to release the pectin and other inclusions. Consequently, this ensures better and faster extraction. 

### 3.5. Fourier Transform Infrared Spectra Analysis of Pectin

**Figure 7:**
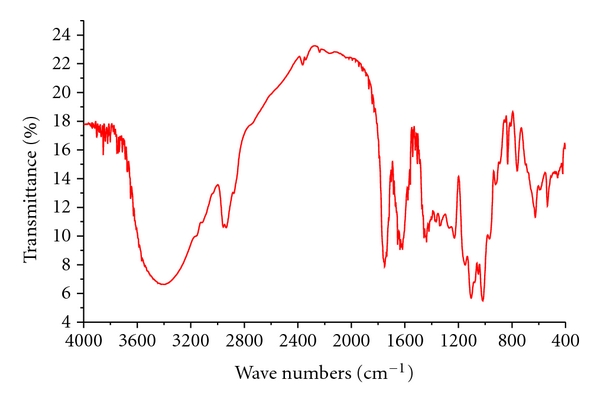
FT-IR spectrum of pectin obtained by the MAE method with ionic liquids.

The FT-IR spectrum of pectin obtained by microwave-assisted extraction with ionic liquids as the solvent is given in Figure 7. The peaks at 2947 cm^−1^ and 2876 cm^−1^ are corresponding to the C-H bond vibration, which are usually covered by O–H group stretching. The peak at approximately 1735 cm^−1^ relates to the C=O bond vibration and indicates the acetyl (COCH_3_) groups in pectin. The characteristic peak at 1626 cm^−1^ is due to the –O– tensile vibration band. The peaks at 1451 cm^−1^, 1370 cm^−1^ and 1337 cm^−1^ represent the C–O–H in the bending vibration. In addition, there is a very weak C–O tensile vibration at 1276 cm^−1^. The peak at 1236 cm^−1^ is asymmetric C–O–C tensile vibration, and indicates the abundance of –O–CH_3 _(methoxyl) groups. The strong peaks at 1108 cm^−1^ and 1021 cm^−1^ are the symmetric C–O–C tensile vibration. From these results, we can conclude that the pectin has been successfully extracted during process. 

## 4. Conclusions 

Ionic liquid solutions have been proposed to be alternative solvent in the microwave-assisted pectin extraction from lemon peels in the present study. The FTIR spectra identify and prove the actual extraction of pectin and the SEM images reveal the peel pulp's loose microstructure after MAE extraction. Under the optimal conditions, the yield of pectin from the microwave-assisted extraction could be 24.68%, which is much higher than the yield from the conventional heating reflux extraction. The optimal extraction time is 8–10 min for the microwave-assisted extraction, which is much shorter compared to 2 h for the conventional heating reflux extraction. Experimental results thus show that the ionic liquid solutions as the solvent are very effective in the microwave-assisted extraction of pectin from lemon peels. 

## Figures and Tables

**Table 1:** Code and level of factors for the trials.

Factor	Coded levels						
	Coded	Uncoded	−1.682	−1	0	1	+1.682
Extraction temperature (°C)	*x*_1_	*X*_1_	58.2	65	75	85	91.8
Extraction time (min)	*x*_2_	*X*_2_	4.6	6	8	10	11.4
Liquid-soild ratio (mL·g^−1^)	*x*_3_	*X*_3_	11.6	15	20	25	28.4

**Table 2:** Experimental designs and the response for the yield of pectin.

Trial No.	Level			Response (%)	
	*x*_1_	*x*_2_	*x*_3_	Observed	Expected
1	−1	−1	1	16.45	16.41
2	0	0	0	21.39	21.86
3	−1	1	1	18.79	19.21
4	0	1.682	0	21.55	21.40
5	1	1	−1	22.32	22.05
6	0	0	0	21.93	21.86
7	−1.682	0	0	15.37	15.34
8	0	0	1.682	21.17	20.82
9	0	0	−1.682	16.88	17.66
10	1.682	0	0	24.54	25.01
11	1	−1	−1	22.04	21.31
12	−1	1	−1	16.58	16.37
13	0	−1.682	0	17.85	18.43
14	1	−1	1	22.32	22.22
15	0	0	0	22.43	21.86
16	1	1	1	24.45	24.51
17	−1	−1	−1	15.48	15.12
18	0	0	0	21.65	21.86
19	0	0	0	21.86	21.86
20	0	0	0	21.95	21.86

**Table 3:** Variance analysis of regression equation.

Source	Sum of Squares	DF	Mean square	*F* value	*P*-value	
Model	157.31	9	17.48	60.62	<0.0001	significant
*x*_1_	112.82	1	112.82	391.28	<0.0001	
*x*_2_	10.67	1	10.67	37.01	0.0001	
*x*_3_	12.01	1	12.01	41.64	<0.0001	
*x*_1_ *x*_2_	0.13	1	0.13	0.46	0.5130	
*x*_1_ *x*_3_	0.074	1	0.074	0.26	0.6231	
*x*_2_ *x*_3_	1.19	1	1.19	4.14	0.0693	
*x*_1_^2^	5.11	1	5.11	17.72	0.0018	
*x*_2_^2^	6.77	1	6.77	23.50	0.0007	
*x*_3_^2^	12.31	1	12.31	42.70	<0.0001	
Residual	2.88	10	0.29			
Lack of fit	2.28	5	0.46	3.79	0.0852	not significant
Pure error	0.60	5	0.12			
Cor total	160.20	19				

Coefficient of variation (CV)  = 3.12; coefficient determination (*R* ^2^) = 0.9820.
